# MetaLook: a 3D visualisation software for marine ecological genomics

**DOI:** 10.1186/1471-2105-8-406

**Published:** 2007-10-22

**Authors:** Thierry Lombardot, Renzo Kottmann, Gregory Giuliani, Andrea de Bono, Nans Addor, Frank Oliver Glöckner

**Affiliations:** 1 Microbial Genomics Group, Max Planck Institute for Marine Microbiology, D-28359 Bremen, Germany; 2 Division of Early Warning and Assessment, Global Resource Information Database – Europe, United Nations Environment Programme, International Environment House, 1219 Châtelaine, Switzerland; 3 Jacobs University Bremen gGmbH, D-28759 Bremen, Germany

## Abstract

**Background:**

Marine ecological genomics can be defined as the application of genomic sciences to understand the structure and function of marine ecosystems. In this field of research, the analysis of genomes and metagenomes of environmental relevance must take into account the corresponding habitat (contextual) data, e.g. water depth, physical and chemical parameters. The creation of specialised software tools and databases is requisite to allow this new kind of integrated analysis.

**Results:**

We implemented the MetaLook software for visualisation and analysis of marine ecological genomic and metagenomic data with respect to habitat parameters. MetaLook offers a three-dimensional user interface to interactively visualise DNA sequences on a world map, based on a centralised georeferenced database. The user can define *environmental containers *to organise the sequences according to different habitat criteria. To find similar sequences, the containers can be queried with either genes from the georeferenced database or user-imported sequences, using the BLAST algorithm. This allows an interactive assessment of the distribution of gene functions in the environment.

**Conclusion:**

MetaLook allows scientists to investigate sequence data in their environmental context and to explore correlations between genes and habitat parameters. This software is a step towards the creation of specialised tools to study constrained distributions and habitat specificity of genes correlated with specific processes.

MetaLook is available at:

## Background

The cost reduction and high-throughput automation of DNA sequencing over the last years have had a profound impact on the field of microbial ecology, giving birth to the field of ecological genomics. Ecological genomics can be defined as the application of genomic sciences to understand the structure and function of marine ecosystems. This field of research is focussed on the investigation of environmentally relevant microorganisms taken from their natural habitats. The sequencing of the genomes of such organisms, especially the new wave of ecological metagenomics, in which DNA sequences are directly retrieved from the environment without prior cultivation, produces huge amounts of new proteins, which theoretically reflect the prominent metabolic processes in the environment [1,2].

Nevertheless, the functional potential coded in the DNA sequences can be successfully interpreted only if considered in their ecological context. Currently, general-purpose DNA databases, as provided by the International Nucleotide Sequence Database Collaboration (INSDC [3]), store only limited environmental contextual (meta-)information with the sequences, if any. Exact geographic origins and the corresponding on-site physical and chemical parameters are rarely found in these databases. This clearly hinders integrated ecological interpretations and limits the extraction of biological knowledge from raw sequence data. With the increasing awareness of this issue [1] and the introduction of new organisms and sample-centric contextual (meta)-data standards, such as those proposed by the Genomic Standards Consortium (GSC) [4,5], this is likely to change in the future. Furthermore, genomic and metagenomic sequence data can be supplemented by information extraction from the literature for proper georeferencing. In parallel, new specialised database architectures and software tools for data visualisation and interpretation are needed [6], enabling the representation of sequence and habitat data in a geographic information system [7,8]. Here we introduce MetaLook, a 3D visualisation software allowing browsing and interpretation of marine sequence data in their ecological context.

## Implementation

### Database server

Genomes and metagenomes from marine environments were selected for import from the NCBI databases [9] into a local PostgreSQL/PostGIS database [10], according to the following criteria: i) the DNA sequence must be of marine bacterial or archaeal origin; ii) sequence quality must be high (i.e. sequencing coverage of at least eight fold); iii) marker and single genes are rejected; and iv) the geographic origin of the DNA sequences must be known precisely (e.g. from the original publication). Lower quality sequences (draft genomes and short metagenomics reads) will be included in future releases.

Geographic locations were stored in our database for accepted DNA samples. Moreover, on-site contextual (meta-)data, such as physical and chemical parameters at the sampling site, were retrieved manually from the original publications and additional web pages when available. This manual curation step is crucial in order to reliably link on-site contextual data to DNA sequences. Moreover, having the exact geographic position for each sample in our database allows the interpolation of environmental parameters from worldwide data sets. Currently, the following global oceanic physical and chemical parameters are integrated into our database from the WOA data set (World Ocean Atlas): temperature, nitrate, phosphate, oxygen and silicate concentration, as well as salinity [11].

### Java 3D-based client

**Figure 1:**
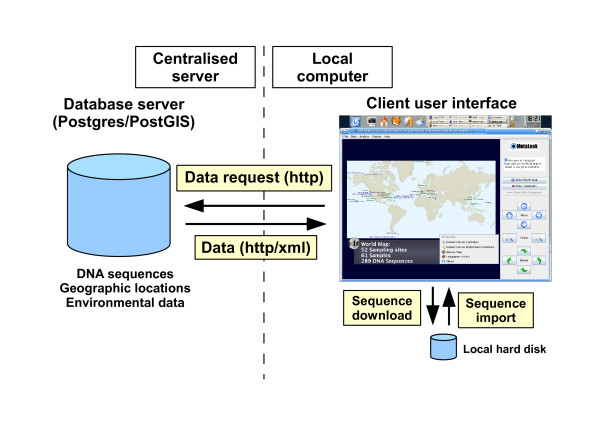
**Client/Server architecture of MetaLook**. The Java3D client runs on a local machine and gets data from the PostgreSQL server through HTTP request in XML format. DNA sequences of interest can be up- and downloaded for further analysis.

**Figure 2:**
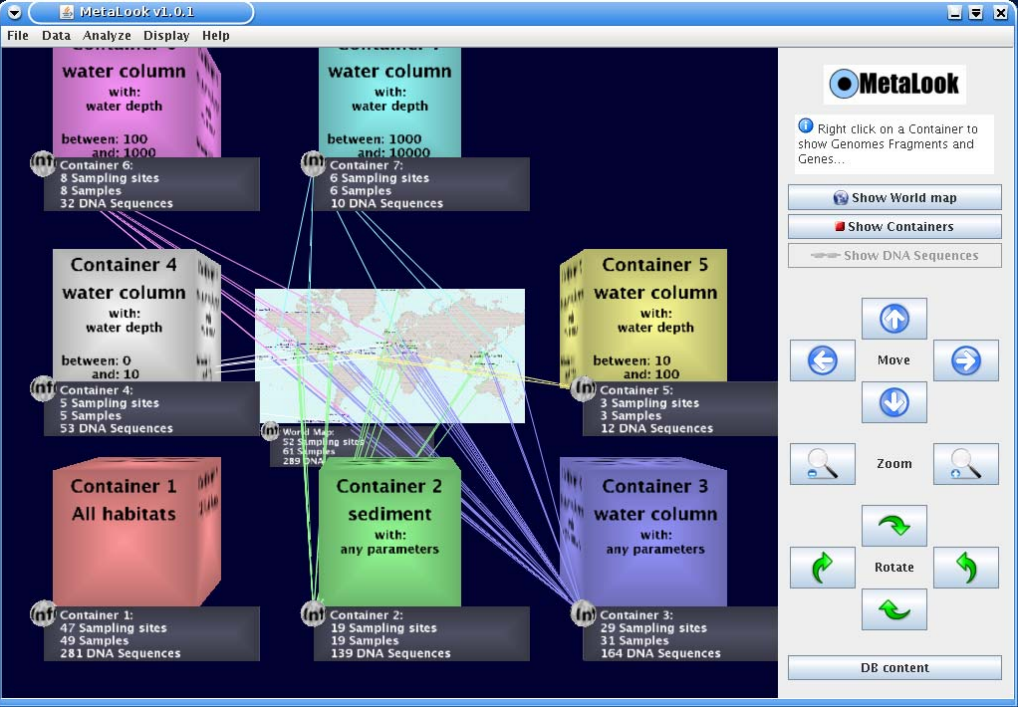
**The *environmental containers *in MetaLook**. DNA sequences of genomes and metagenomes can be sorted into 3D containers according to habitat information such as e.g. water column vs. sediments, depth profile or physical-chemical parameters. The geographic origins of the DNA sequence samples in each container are shown on the world map.

**Figure 3:**
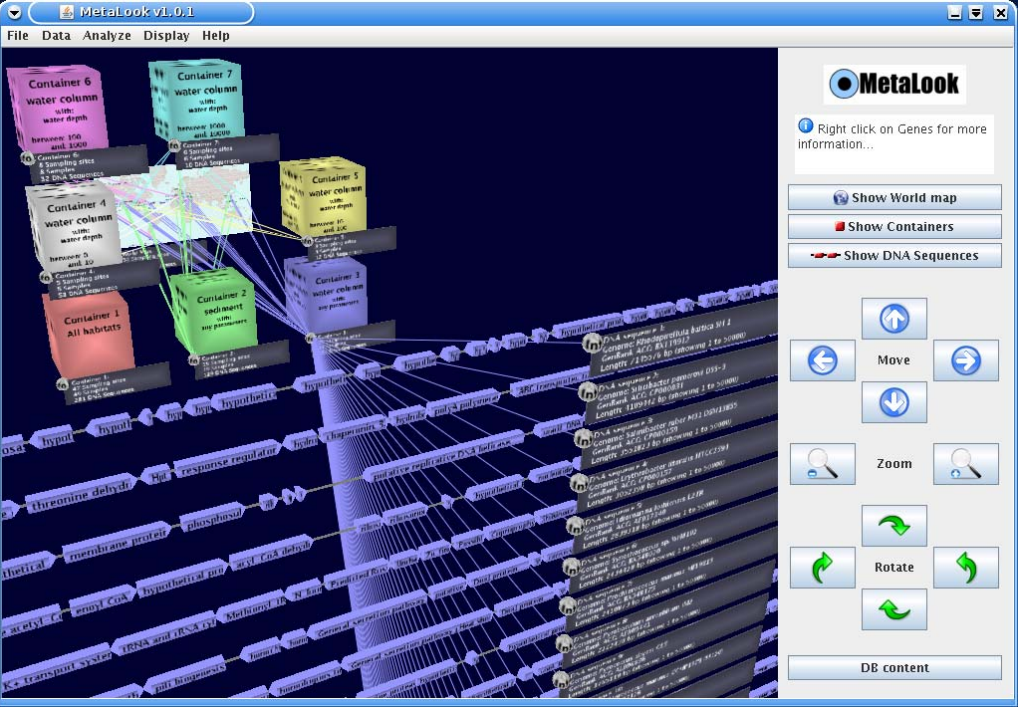
**Displaying (meta)-genomes and genes in MetaLook**. Each container can be opened to display the DNA sequence and the genes of each genome and metagenomic fragment. Genes can further be selected for download or analysis.

The MetaLook interface is a locally running client based on the Java 3D API [12], started using the Java Web Start technology from the megx.net data portal [7]. The starting point of the interface is a 3D workbench displaying a world map with the sampling sites of genomic and metagenomic studies available in our database (Fig. 1). The 3D approach allows displaying larger amounts of data and interconnections than a classical 2D visualisation [13]. Within MetaLook, the user can sort the corresponding DNA sequence data into so-called *environmental containers*, which are flexible entities grouping data according to specific criteria, such as habitat types, ocean water depth or physical and chemical parameters. This custom data classification allows the user to define ecological niches according to specific biological questions (Fig. 2). The DNA sequence fragments grouped into the containers can be visualised on the workbench for browsing and comparing (Fig. 3). Moreover, each container can be searched for specific genes based on their annotations. Search results are shown graphically in their genomic context. The DNA or protein sequence of each gene can be displayed or easily downloaded from the database. All DNA sequences in a container can be downloaded in batch mode. Custom sequences can be imported into the MetaLook interface in FASTA format.

### BLAST against *environmental containers*

**Figure 4:**
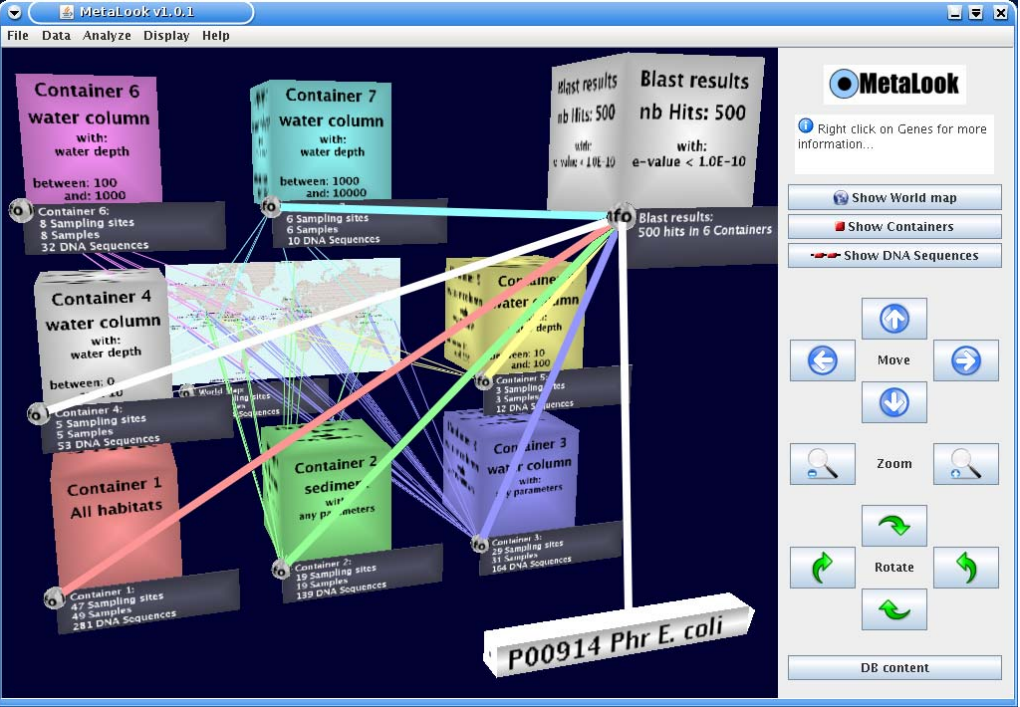
**Study of the habitat-specificity of a gene**. Here, the gene encoding a photolyase (foreground) shows BLASTP hits in the top layers of the ocean, as expected, but also some unexpected hits in the deep sea (container 7).

**Figure 5:**
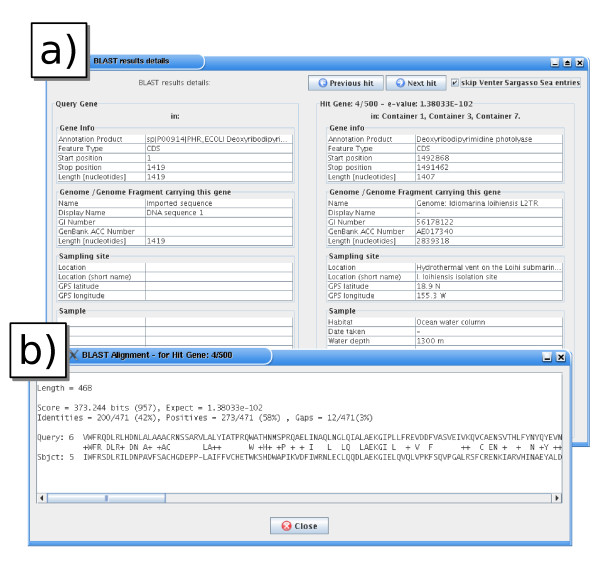
**Study of the habitat-specificity of a gene (habitat parameters)**. a) Information for an unexpected BLASTP hit of the photolyase gene from figure 4 with a sequence originating from a deep-sea sample; b) BLASTP sequence alignment for the corresponding sequences.

Any protein encoding gene from our georeferenced database or user-imported sequences can be used as a query for a BLASTP run [14] against the genes grouped into user-defined *environmental containers*. The BLASTP analysis is started from the MetaLook interface (client) and runs on the centralised server. The results are shown graphically using 3D connectors between the query gene and the containers with sequence matches (Fig. 4). This representation reveals the distribution of similar genes in the user-defined habitats. The results are saved in a result panel for detailed investigation, showing the habitat parameters of each match, the corresponding BLASTP *e*-value, and sequence alignment (Fig. 5a, b).

### Comparison to other programs

Some interesting DNA sequence tools making use of 3D are currently available. Sockeye is a 3D environment for comparative genomics allowing simultaneous visualisation of the annotations of different eukaryotic organisms [15]. The Correlogo server is a tool to display DNA sequence alignments using 3D sequence logos [16]. The Walrus graph visualisation tool allows visualisation of very large phylogenetic trees in a hyperbolic space [17]. These examples show the benefits of advanced visualisation tools for DNA research and the management of large data sets. However, within this context, MetaLook is unique in its orientation toward environmental genomics, geographic and contextual data integration.

## Results and Discussion

The MetaLook interface allows the sorting of sequence data according to sampling sites and habitat parameters, with respect to targeted biological questions. The distribution of genes in the environment is revealed using the BLAST algorithm with a selected query gene against other sequences sorted in *environmental containers*. The following examples illustrate some expected and unexpected habitat distributions of genes in the environment using the MetaLook interface.

### Methanogenesis genes (mch and mcr)

In microorganisms, methanogenesis is a form of microbial anaerobic respiration leading to the formation of methane. Recent experimental and genomic data support the hypothesis that anaerobic oxidation of methane (AOM) is using a reverse-methanogenesis pathway [18-20]. Such biochemical processes are crucial in the environment, as methane is an important greenhouse gas contributing to global warming. One of the key genes of methanogenesis and AOM is mcr, encoding a methyl-coenzyme-M reductase (Mcr). The distribution of mcr in the environment was visualised by MetaLook with the following steps: i) predefined *environmental containers *were created from the world map, grouping sediment and water samples by depth (Fig. 2); ii) a text search for the gene "mcr" was performed; iii) Mcr protein sequences (e.g. McrB, [Genbank: AAB98847]) were blasted against all containers (BLASTP, *e*-value cut-off 10^-10^). The results show that within the georeferenced marine bacteria and archaea currently available in our database, genes encoding Mcr are only found in sediments. Although expected, this observation shows that mcr genes are habitat specific, which is consistent with the strictly anaerobic nature of methanogenesis and the AOM process.

Another key gene of the methanogenesis and AOM processes is mch, encoding a methenyl-tetrahydromethanopterin cyclohydrolase (Mch). Interestingly, this gene was reported in some proteobacteria and planctomycetes, where an archaea-like C1 metabolism appears to be present [21]. Following the same procedure used for mcr (see above) revealed, as expected, that the mch gene is not only present in genomes and metagenomes originating from sediments, but is also found in the genome of at least one sea water column bacterium, the planctomycete *Rhodopirellula baltica *SH 1^T ^from the Baltic Sea [22] (e.g. [GenBank: CAD74990]). Furthermore, this analysis showed that mch is also found in the high-throughput metagenomics data set of the Sargasso Sea [23], suggesting an even more widespread distribution of this gene in the environment. Hence, the analysis of the habitat specificity of mcr and mch revealed differential environmental distribution of genes relevant for major biochemical processes involved in the global cycling of carbon.

### Photolyase gene (phr)

Solar UV-light induces pyrimidine dimers in genomic material, leading to enhanced mutation rates. Photolyases are proteins involved in a light-dependant, single-step DNA repair mechanisms, which protect microorganisms against this destructive effect [24]. Comparative analysis of the genomes of three *Prochlorococcus marinus *strains, one of the most abundant phototrophic prokaryote in the ocean, previously reported the presence of photolyase encoding genes (phr) in the high-light ecotype, and its absence in the low-light ecotypes (water depth: 5 m and 120/135 m, respectively) [25]. This finding suggests that for this particular species, the phr gene is lost if an organism is exposed to little or no UV-light. As no DNA pyrimidine dimers should form where no UV-light stress occurs, the phr gene is not expected in the deep layers of the ocean.

To systematically test the occurrence of the phr gene in the marine environment, a phr gene with experimental evidence (*Escherichia coli *K-12, [Swiss-Prot: P00914]) was imported into the MetaLook interface and searched against predefined environmental containers with the BLASTP algorithm (*e*-value cut-off 10^-10^). Some sequence hits in the top layers of the ocean were found, as expected (e.g. *Prochlorococcus marinus *MED4, [Genbank: CAE18744] and *Rhodopirellula baltica *SH 1^T^, [Genbank: CAD77347]). Moreover, unexpected sequences from deep-sea water (hot vent) and coastal sediments were also hit by this analysis (*Idiomarina loihiensis *L2TR, [GenBank: AAV82228] and *Hahella chejuensis *KCTC 2396, [GenBank: ABC28582]) [26,27] (Fig. 3). These genes are likely to be functional, with full-length BLASTP alignments and excellent statistical support, with *e*-values below 10^-100 ^(Fig. 4a, b). Such unexpected occurrence of genes encoding photolyases in these environments might be explained by: i) the presence of allochthonous organisms [28], ii) residual phr genes awaiting deletion in organisms recently adapted to deep-sea or sediment environments, or iii) the possible need for protective mechanisms against geothermal light, even if the dominant wavelengths are not in the UV range [29].

### Future work

The availability of worldwide physical and chemical parameters linked to DNA sequences opens the way to multivariate analysis. This approach will be crucial as more georeferenced genomic and metagenomic samples become available. The integration of low quality sequences (e.g. single reads from metagenomics) and biodiversity markers (e.g. ribosomal RNA genes) in our geographic-centric system is also a follow-up perspective.

## Conclusion

Marine ecological genomics is an emerging field of research but available high quality and accurately georeferenced sequence data are still sparse compared to the natural habitat and organism diversity. Therefore, the observed absence of genes in particular habitats may reflect a mere gap in the database coverage. However, with the use of appropriate software tools, common knowledge can be easily confirmed and unexpected findings can be obtained for further investigation, as shown here with the example of a light-dependant gene present in the deep-sea. As more sequences with rich contextual (meta-) data from marine genome and metagenome projects are released, the accuracy and reliability of correlations between gene occurrence and habitat parameters will continuously improve. Targeted studies of gene distribution in the environment are greatly facilitated by our specialised databases and software tools presented here, offering an advanced software workbench for biologists.

## Availability and requirements

Project name: MetaLook

Project home page: 

Direct download and installation (Java web start): 

Operating systems: Windows or Linux.

Programming language: Java.

Other requirements: Java JRE 1.5 or higher, 3D card recommended.

License: license-free.

Any restrictions to use by non-academics: MetaLook may not be sold or bundled with any type of commercial application.

## List of abbreviations used

mcr/Mcr: methyl-coenzyme-M reductase gene/protein.

mch/Mch: methenyl-tetrahydromethanopterin cyclohydrolase gene/protein.

phr/Phr: photolyase gene/protein.

FP6: the Sixth Framework Programme of the European Union.

NEST: new and emerging science and technology.

## Competing interests

The author(s) declares that there are no competing interests.

## Authors' contributions

TL designed and implemented MetaLook, the initial version of the underlying database and integrated the genomic data. RK designed and implemented the current version of the underlying database and integrated the metagenomic data. GG, AB and NA performed WOA data set integration and interpolations. FOG is leading the EU-project MetaFunctions, gave advise for software development, and has made revisions and contributions to the manuscript.
